# Laminin 521 maintains differentiation potential of mouse and human satellite cell-derived myoblasts during long-term culture expansion

**DOI:** 10.1186/s13395-016-0116-4

**Published:** 2016-12-13

**Authors:** Christopher M. Penton, Vasudeo Badarinarayana, Joy Prisco, Elaine Powers, Mark Pincus, Ronald E. Allen, Paul R. August

**Affiliations:** 1 Discovery Biology, Tucson Innovation Center, Icagen, Oro Valley, AZ 85755 USA; 2 Discovery Biology, Tucson Innovation Center, Sanofi, Oro Valley, AZ 85755 USA; 3 School of Animal and Comparative Biomedical Sciences, University of Arizona, Tucson, AZ 85721 USA

**Keywords:** Satellite cell, Myoblast expansion, Stem cell, Laminin 521, Cell therapy

## Abstract

**Background:**

Large-scale expansion of myogenic progenitors is necessary to support the development of high-throughput cellular assays in vitro and to advance genetic engineering approaches necessary to develop cellular therapies for rare muscle diseases. However, optimization has not been performed in order to maintain the differentiation capacity of myogenic cells undergoing long-term cell culture. Multiple extracellular matrices have been utilized for myogenic cell studies, but it remains unclear how different matrices influence long-term myogenic activity in culture. To address this challenge, we have evaluated multiple extracellular matrices in myogenic studies over long-term expansion.

**Methods:**

We evaluated the consequence of propagating mouse and human myogenic stem cell progenitors on various extracellular matrices to determine if they could enhance long-term myogenic potential. For the first time reported, we comprehensively examine the effect of physiologically relevant laminins, laminin 211 and laminin 521, compared to traditionally utilized ECMs (e.g., laminin 111, gelatin, and Matrigel) to assess their capacity to preserve myogenic differentiation potential.

**Results:**

Laminin 521 supported increased proliferation in early phases of expansion and was the only substrate facilitating high-level fusion following eight passages in mouse myoblast cell cultures. In human myoblast cell cultures, laminin 521 supported increased proliferation during expansion and superior differentiation with myotube hypertrophy. Counterintuitively however, laminin 211, the native laminin isoform in resting skeletal muscle, resulted in low proliferation and poor differentiation in mouse and human cultures. Matrigel performed excellent in short-term mouse studies but showed high amounts of variability following long-term expansion.

**Conclusions:**

These results demonstrate laminin 521 is a superior substrate for both short-term and long-term myogenic cell culture applications compared to other commonly utilized substrates. Since Matrigel cannot be used for clinical applications, we propose that laminin 521 could possibly be employed in the future to provide myoblasts for cellular therapy directed clinical studies.

**Electronic supplementary material:**

The online version of this article (doi:10.1186/s13395-016-0116-4) contains supplementary material, which is available to authorized users.

## Background

Satellite cells are the major effector cell responsible for eliciting muscle regeneration. The satellite cell name was conferred based on its identification on the periphery of the myofiber characterized by very little cytoplasm and a prominent nucleus. In this position, satellite cells remain in an inactive quiescent state characterized and regulated by the transcription factor Pax7 [1]. Once activated in response to muscle damage, satellite cells up-regulate the transcription factor MyoD and enter the cell cycle as transit-amplifying myoblasts [2]. Once they reach sufficient numbers, myoblasts exit the cell cycle, increase expression of myogenin, and differentiate to form multinucleated myotubes through the process of cellular fusion [3, 4]. These myotubes form the building blocks for functional, contractile muscle fibers.

In vivo, satellite cell activity is regulated by what is referred to as the satellite cell “niche,” an extracellular environment between the muscle fiber and the basal lamina. In this location, extracellular matrix adhesion proteins influence satellite cell activity in their quiescent and activated states. Laminin 211, a heterotrimeric complex composed of α2, β1, and λ1 chains, is a primary component of the niche interacting with the satellite cell via the integrin α7β1 complex [5]. Following activation, multiple extracellular matrix (ECM) proteins are induced in the skeletal muscle including fibronectin (FN), collagen 1, and collagen 3. Fibronectin binds to satellite cells by interaction with integrin α4β1 and integrin α5β1 complexes [6, 7]. Fibronectin is up-regulated in the niche acting via Wnt7a and functions to expand the stem cell pool of satellite cells and maintain stem cell numbers [8, 9]. Laminin α5 is an additional laminin isoform localized to regenerating myofibers that is up-regulated specifically during murine skeletal muscle regeneration and in human dystrophic muscle [10, 11]. However, functional experiments have not been performed to examine the satellite cell-laminin α5 relationship in detail. Although satellite cell laminin α5-integrin studies have not been examined previously, experimental studies have revealed that laminin α5 contains the greatest number of integrin-binding sites that include those for α3β1 (2×), αVβ3, α6β1, α6β4, and α7β1 [12–16].

The expansion of satellite cells and myoblasts is a critical component for both neuromuscular drug discovery and cell therapy applications. In both of these applications, methods are required to provide large-scale expansion of myoblast cells while maintaining high differentiation capacity and diminished spontaneous differentiation. Unfortunately however, comprehensive analysis of myoblasts has not been performed after extended passaging in vitro; the majority of experimental studies have been performed on satellite cells and myoblasts that were freshly isolated due to the challenges of expansion in vitro. Moreover, our attempts to perform large expansion of satellite cell and myoblasts have been challenged due to the progressive loss of differentiation over time in culture (unpublished data). This challenge may hinder scaling of myogenic cells for multiple applications including high-throughput screening and cell therapy. One potential reason for this phenomenon may be the use of biologically irrelevant cell substrates during myoblast expansion. For instance, myoblast are often cultured on a variety of ECM substrates including laminin 111, fibronectin, gelatin, collagen I, and Matrigel (MG) [17–21]. Although laminin is commonly utilized in myogenic cell culture, the form commonly employed is laminin 111 which is composed of α1, β1, and λ1 chains; laminin structure is illustrated in Additional file 1: Figure S1. Laminin 111 is not expressed in adult skeletal muscle and differs from laminin 211, the satellite cell niche component, containing a different alpha chain [10]. In addition, other substrates mentioned, MG, gelatin, and collagen I are not associated with the satellite cell niche. Moreover, since Matrigel is animal-derived, myogenic cells cannot be expanded on Matrigel for clinical trials or future cell therapeutic applications. Nevertheless, while short-term testing has been performed previously, long-term myoblast expansion has not been compared between cellular substrates. In this study, we present both short-term and long-term expansion analysis of primary mouse and human myoblast cultures on both commonly used substrates laminin 111 and MG, as well as previously untested but biologically relevant laminin 211 (α2, β1, λ1) and laminin 511/521 (α5, β1, λ1; α5, β2, λ1) substrates.

## Methods

### Isolation and culture of murine satellite cells

Primary murine satellite cells were isolated from the tibialis anterior and quadriceps muscles from 12-week-old DBA/2J male mice. Dissected muscles were minced with scalpel blades and digested in DMEM/F12 (Life Technologies, 1:1 mixture) containing 2% collagenase II (Worthington Biochemicals) and 1.2 U/ml dispase (Worthington Biochemicals) with 2.5 mM CaCL_2_. Digestions were incubated at 37 °C for 1 h with trituration and mixing every 15 min. The cells were filtered through 100 and 40-μM cell strainers (BD). The cells were pelleted by centrifugation for 5 min at 300×*g*. The cells were resuspended in FACS staining buffer (DMEM/F12/0.5% BSA/25 mM HEPES) and distributed in 200-μl aliquots into staining tubes. The cells were blocked using anti-CD16/CD32 antibody (Ebioscience) at 1:100 dilution for 10 min on ice. The cells were stained with the following antibodies on ice for 30 min: CD31-FITC (1:50, Ebioscience, 390) CD45-FITC (1:50, Ebioscience, 30-F11), PDGFRα-BV421 (1:40, BD, APA5), Sca1-BV605 (1:100, BD, D7), and integrin α7 (1:400, Ablab, R2F2). The cells were washed twice with sort buffer (HBSS/0.5% BSA/25 mM HEPES) including centrifugation for 5 min at 300 g. Compensation controls were prepared using Ultracomp beads (Ebioscience). Single only bead controls were stained in 100 μl with 2 μl of each antibody for 15 min at room temperature. The beads were washed once with sort buffer and resuspended in sort buffer. Compensation was calculated using single-stained and unstained bead controls with FACS DIVA compensation wizard. Gating was determined by using fluorescence minus one plus isotype controls. Dead cells were gated out using propidium iodide (Life Technologies).

### ECM coating and culture

Laminins including laminin 111, laminin 211, laminin 332, laminin 411, laminin 421, laminin 511, and laminin 521 are human recombinant isoforms obtained from Biolamina (Sweden). Laminins are diluted at a concentration of 10 μg/ml in HBSS with calcium and magnesium and coated overnight at 4 °C. Growth factor reduced Matrigel (Corning) was diluted 1:5 with DMEM/F12 media and thin coated by covering plastic, removing excess, and drying Matrigel for 20 min at 37 °C.

For initial characterization, the cells were plated at a density of 2000 cells per well in a 96-well format in DMEM/F12/20% FBS/Primocin (Live Technologies/InvivoGen) with 10 ng/ml mouse FGF-2 (R&D). Media were replaced after 5 days and refreshed every 3 days afterwards. To induce differentiation at day 8, the media were switched to differentiation media (DM), DMEM/F12/5% HI-HS/Primocin, and maintained until day 11.

For long-term growth, the cells were plated at a density of 10,000 cells per well in a 6-well format. The cells were grown in growth media as previously described and refreshed every 3–4 days with growth media and 10 ng/ml FGF-2. Cells were split using Accutase and maintained on the same substrate for six to eight passages. To assay differentiation, the cells were split using Accutase and seeded in a 96-well format at a density of 4000 cells per well. The cells were grown in GM for 5 days, and then switched to DM for an additional 5 days. For ECM substitution experiments, the cells were thawed, expanded, and passaged twice before analysis. At the second passage, the cells were transferred to a 96-well plate containing four of the ECM substrates (laminin 111, laminin 211, laminin 521, and MG). The cells were grown and differentiated similarly to our previously mentioned long-term growth procedure.

### Immunocytochemistry and imaging

Immunostaining was performed in black Corning 96-well plates. For myosin heavy chain (MHC) staining, the cells were fixed using Cytoperm/Cytofix for 15 min at room temperature. The cells were rinsed twice and then subsequently blocked using 10% HI-HS/0.1% Triton for 1 h at room temperature. The cells were stained with MHC-Alexa488 antibody at 1:100 overnight at 4 °C. The cells were rinsed four times with PBS and stained with Hoechst to identify nuclei. Images were acquired using a ×10 objective on a Cellomics ArrayScan. Analysis was performed using the Cellomics HCS Studio Version 6.5 software analyzing MHC-positive cells containing two or more nuclei. The software algorithm used was the “myotube formation” package using dynamic thresholding, three sigma or isodata, for myotube identification.

For Pax7/MyoD staining, the cells were fixed using foxp3/ki67 nuclear fixation buffer (Ebioscience) for 15 min at room temperature. The cells were rinsed twice and blocked with Block Aid (Life Technologies) for 1 h at room temperature. Pax7 (1:50, R&D) and MyoD (1:50, 5F11, Millipore) were coincubated overnight at 4 °C in Block Aid. The cells were rinsed three times, and secondary antibodies (donkey anti-mouse Alexa488, donkey anti-rat Alexa647; 1:200) were incubated for 1 h at room temperature. The cells were rinsed four times and stained with Hoechst for nuclei identification. Images were acquired using a ×20 objective on a Cellomics ArrayScan. Analysis was performed using the nuclear colocalization algorithm (Cellomics HCS Studio 6.5) analyzing proportion of Pax7 or MyoD-positive nuclei.

### Integrin FACS analysis

Passage 8 mouse myoblasts were split using Accutase, collected and centrifuged at 300×*g* for 5 min, and resuspended in FACS staining buffer. The cells were blocked with FC block (BD biosciences) at 1:50 for 10 min on ice. Afterwards, the cells were stained with the following PE-conjugated antibodies: integrin alpha1 (BD 562115) at 1:40, integrin alpha2 (Ebioscience 12-5971-81) at 1:40, integrin alpha3 (R&D FAP2787P) at 1:10, integrin alpha4 (Ebioscience 12-0492-81) at 1:20, integrin alpha5 (BD 553930) at 1:40, integrin alpha6 (Ebioscience 12-0495-81) at 1:200, integrin alpha7 (Ablab) at 1:200, integrin alphaV (Ebioscience 12-0512-82) at 1:50, integrin beta1 (Ebioscience 12-0291-81) at 1:20, integrin beta2 (Ebioscience 12-0181-81) at 1:20, integrin beta3 (Ebioscience 12-0611-81) at 1:40, integrin beta4 (R&D FAB4054P) at 1:20, and integrin beta5 (Ebioscience 12-0497-41) at 1:20. The cells were stained for 30 min on ice followed by two washes in FACS stain buffer. The cells were resuspended in 300 μl of FACS buffer and analyzed on the FACSAria II. Gating was set according to negative unstained and isotype control Rat IgG2a K-PE (Ebioscience 12-4321-81).

### Human myogenic cell isolation

Post-mortem non-diseased skeletal muscle gracillis tissue was obtained through Asterand. The muscle was trimmed of fat and connective tissue. The tissue was minced for approximately 10 min. The tissue was digested using Collagenase II (Worthington Biochemicals) and Dispase (Worthington Biochemicals), for approximately 75 min at 37 °C. Digestions were performed in gentleMACS™ Dissociators. The tissue was pulsed every 15 min. Following digestion, the cells were strained through 100-, 70-, and 30-μM cells strainers (Miltenyi), respectively. The cells are resuspended in approximately 200 μl of MACS stain buffer (Miltenyi). The cells are stained for 1 h on ice with the following antibodies: CD11b-FITC (Miltenyi Biotec, Catalog Number: 130-081-201), CD31-FITC (Miltenyi Biotec, Catalog Number: 130-092-654), CD45-FITC (Miltenyi Biotec, Catalog Number: 130-080-202), CD34-APC (BD Biosciences, Catalog Number: 560940), and CD56-PE (Miltenyi Biotec, Catalog Number: 130-090-755). Afterwards, the cells were rinsed twice and subsequently incubated with anti-FITC microbeads (Miltenyi Biotec, 130-048-701) for 30 min on ice followed by two washes. Afterwards, the cells were passed through a Miltenyi magnetic depletion column. The column binds magnetically labeled FITC+ cells (CD31, CD45, CD11b) while allowing FITC− cells to flow through. The cells move passively through the column into a collection tube. Afterwards, the cells were centrifuged, resuspended in FACS buffer, and FACS sorted (FACS ARIA II) for CD56+, CD34−, CD45−, CD31−, and CD11b− cells. Myogenic cells were grown in growth media DMEM/F12 (Gibco) supplemented with 20% FBS (Gibco)/Primocin and 10 ng/ml human FGF2 (R&D). For differentiation of human cells, cells were seeded at a density of 16,000 cells per well in a 96-well format. After 3 days, half of the media was replaced with differentiation media consisting of DMEM/F12 supplemented with 5% HS-HI (Gibco) and Primocin. Afterwards, half of the media was replaced every other day until day 11 when the cells were fixed with Cytoperm/Cytofix (BD).

### Statistics

Statistics for multiple comparisons were conducted using one-way ANOVA with Bonferroni correction. Significance is annotated as less than .05 (*), less than .01 (**), less than .001 (***), and less than .0001 (****). All comparisons were conducted using laminin 521 as control. Significance for myotube nuclei distribution was determined using linear regression. Statistical calculations were conducted using Graphpad Prism 6.

## Results

### ECM influences myogenic potential

**Fig. 1:**
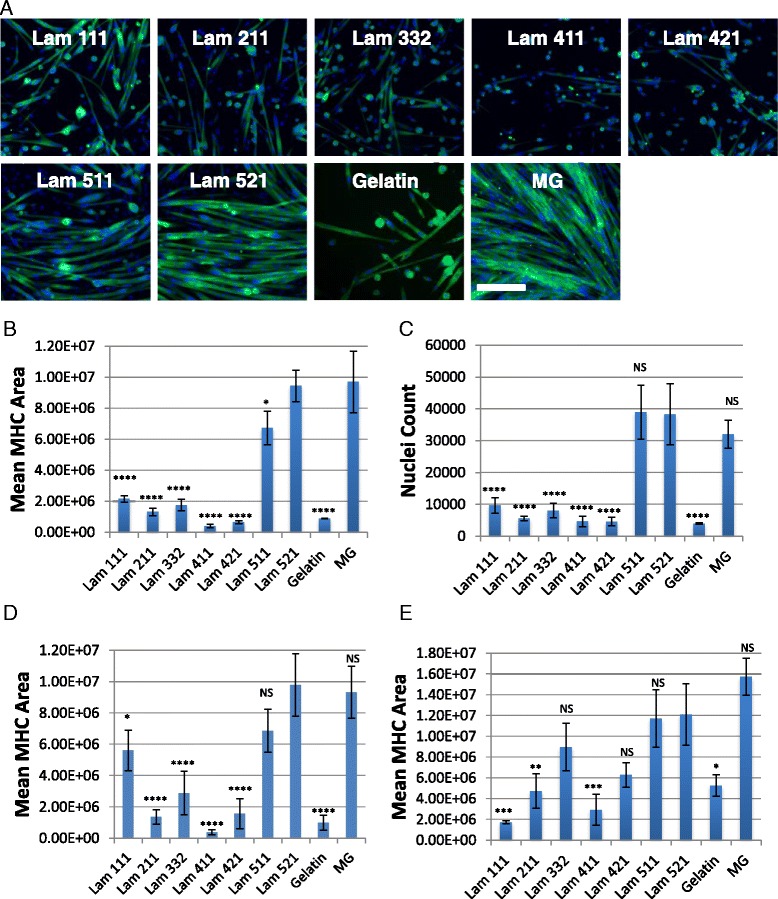
Laminin 521/511 and MG support superior differentiation and proliferation in freshly isolated satellite cells. **a** Differentiation of freshly isolated satellite cells (Dba/2J), myosin heavy chain (*green*), and Hoechst (*blue*). **b** Quantification of differentiation via mean MHC calculated area (Dba/2J). **c** Quantification of total nuclei per well via Hoechst nuclei count (Dba/2J). Quantification of differentiation via MHC calculated area from freshly isolated C57/BL6 (**d**) and C57/BL10 (**e**). *Error bars* represent means ±S.D. *Scale bar* represents 200 μM. *N* = 3 biological replicates per group. Statistical significance was determined by one-way ANOVA with Bonferroni correction. Significance is annotated as less than .05 (*), less than .01 (**), less than .001 (***), and less than .0001 (****). All comparisons were conducted using laminin 521 as control

To compare the activity of freshly isolated mouse satellite cells, we FACS sorted Integrinα7+/PDGFRα–/Sca1−/CD31−/CD45− cells (Additional file 2: Figure S2) and plated on ECM substrates including laminin 111, laminin 211, laminin 332, laminin 411, laminin 421, laminin 511, laminin 521, gelatin, and growth factor reduced MG (Fig. 1a). We observed a striking increase in proliferation resulting in a three- to fourfold increase in cell number on laminin 511, laminin 521, and MG compared to all other substrates (Fig. 1a, c). In addition, the cells expanded on laminin 511, laminin 521, and MG showed dramatically enhanced differentiation as quantified by MHC-positive area (Fig. 1b). On the other hand, cells on laminin 111 differentiated moderately while cells on laminin 211, laminin 332, laminin 411, and laminin 421 differentiated poorly (Fig. 1a, b). Additionally, myotubes formed on laminin 521 and MG visually appeared to be wider in appearance and overall more robust. Cells differentiated on laminin 521 appeared to be more organized in comparison and more mature as we noted the nuclei in laminin 521 cultures evenly spaced and distributed while MG myotubes nuclei had a clustered appearance and myotubes appeared less organized. To extend these results beyond the Dba mouse model, we repeated our differentiation experiments on C57/BL6 and C57/BL10 satellite cells. Consistent with the Dba satellite cell results, cellular differentiation is consistently increased on laminin 511, laminin 521, and MG compared to all other substrates (Fig. 1d, e).

**Fig. 2:**
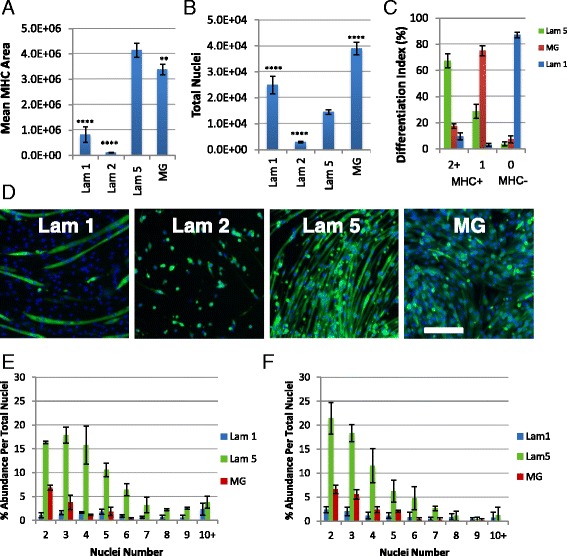
Laminin 521 is the only substrate capable of maintaining differentiation after eight passages in Dba satellite cell-derived myoblasts. **a** Quantification of differentiation via MHC area calculation. **b** Quantification of total nuclei per well via Hoechst nuclei count. Statistical significance was determined by one-way ANOVA with Bonferroni correction. *N* = 3 technical replicates per group from one biological sample. Significance is annotated as less than .01 (**), and less than .0001(****). All comparisons were conducted using laminin 521 as control. **c** Differentiation index, calculation of proportion of MHC+ myotubes containing two or more nuclei, MHC+ myocytes containing a single nuclei, and proportion of MHC− cells. **d** Immunostaining for MHC (*green*) and Hoechst (*blue*). *Scale bar* represents 200 μM. *N* = 4 wells per group. **e**, **f** Multi-nucleation index quantifying the number of nuclei per myotube as a proportion of total nuclei at passage 6 (**e**) and passage 8 (**f**). Statistical significance for **e**, **f** was determined by linear regression. The distribution of laminin 521 was found to be significantly different, *p* < .001, compared to laminin 111 and MG

**Fig. 3:**
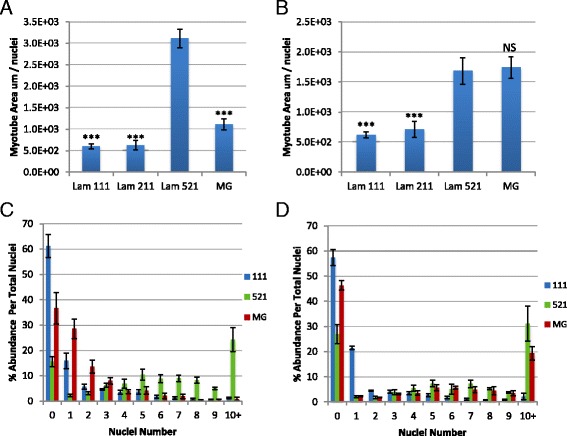
Laminin 521 supports robust differentiation in C57/BL6 and C57/BL10 backgrounds after eight passages. Quantification of differentiation via MHC area calculation in proportion to total nuclei for BL6 (**a**) and BL10 (**b**). **c**, **d** Multi-nucleation index quantifying the number of nuclei per myotube as a proportion of total nuclei in BL6 (**c**) and BL10 (**d**) cultures at passage 8. Statistical significance for **a**, **b** was calculated using one-way ANOVA with Bonferroni correction. *N* = 3 technical replicates per group. Significance is annotated as less than .001 (***). All comparisons were conducted using laminin 521 as control. Statistical significance for **c**, **d** was calculated using linear regression. The distribution of laminin 521 was found to be significantly different, *p* < .001, compared to laminin 111 and MG

While satellite cells typically have strong differentiation potential when freshly isolated, no study to our knowledge has evaluated a variety of ECMs to expand satellite cell-derived myoblasts to maintain their stem cell and differentiation potential after long-term culture. Therefore, we selected laminin 111 and MG due to their common usage in the literature, as well as laminin 211 and laminin 521 due to their expression in vivo*.* Additionally, we selected laminin 521 over laminin 511 due to the observed performance benefit in our short-term study in Fig. 1. Dba cells were grown for six to eight passages and then assayed for proliferation and differentiation. Similar to our short-term results, we found significant differences in differentiation among different ECMs, and these differences appear to be amplified over the long term. Laminin 111 displayed significant proliferation but differentiated minimally (Fig. 2a, d). While the myotubes formed on laminin 111 were fairly large in size, the majority of the cells in culture were negative for MHC (Fig. 2c). Laminin 211 on the other hand performed similarly to the fresh analysis from Fig. 3 where cells expanded at a very slow rate and failed to differentiate (Fig. 2a, d). Laminin 521 and MG both were the only substrates that supported extensive myogenic differentiation, as assayed by MHC-positive area after culture in DM (Fig. 2a, d). However, while laminin 521 and MG have similar MHC area percentages, cells on laminin 521 form more multinucleated myotubes, defined as myotubes containing two or more nuclei, compared to cells on MG (Fig. 2c). Moreover, MG cells up-regulate MHC but fail to fuse significantly remaining in a myocyte stage resulting in approximately 70% of the cells expressing MHC but only containing one nucleus (Fig. 2c). On the other hand, 70% of laminin 521 assayed cells contain two or more nuclei (Fig. 2c). To further quantify myogenic differentiation, we performed an in-depth multi-nucleation index to quantify the number of nuclei per myotube proportional to the total nuclei of myotubes on laminin 111, laminin 521, and MG. We determined at both passage 6 and passage 8 that laminin 521 myotubes contained increased proportions of nuclei per myotube compared to MG and laminin 111 (Fig. 2e, f). Strikingly, laminin 521 myotubes contained a broad increase in the proportion of nuclei per myotube over the entire distribution of myotubes ranging from two to ten nuclei per myotube (Fig. 2e, f). Overall, these results reveal that laminin 521 may be a superior substrate for expanding myogenic cell cultures over long-term passage while maintaining excellent differentiation.

In order to confirm laminin 521 activity on additional background strains over long-term passaging, we cultured C57BL/6J (BL6) and C57BL/10ScNJ (BL10) primary myoblasts to eight passages and assayed for differentiation performance. Similar to previously discussed findings, laminin 521 expanded myoblasts from BL6 and BL10 strains robustly differentiated into multinucleated myotubes at a significantly higher efficiency compared to laminin 111 and laminin 211 (Fig. 3a, b). MG performance varied substantially between BL6 and BL10 expanded cells; while BL6 cells exhibited poor differentiation, BL10 cells exhibited a similar amount of MHC area per nuclei compared to laminin 521. Multi-nucleation indexes were next calculated for BL6 and Bl10 cells on each of the four substrates including laminin 111, laminin 211, laminin 521, and MG. In BL6 cultures, laminin 521 cells show a significant shift of nuclei distribution towards myotubes containing five or more nuclei while laminin 111 and MG cultures were enriched for myotubes containing one to four nuclei in MHC-positive cells (Fig. 3c). In addition, laminin 111 and MG contained a significantly higher number of undifferentiated MHC-negative cells in BL6 cultures (Fig. 3c). In BL10 cultures, laminin 521 and MG performed similarly regarding myonuclei distribution although laminin 521 contained approximately 10% increased proportion of myotubes containing 10 or more nuclei and laminin 521 contained approximately 20% reduced proportions of MHC-negative cells (Fig. 3d). Overall, these results confirm laminin 521 promotes the most optimal and consistent differentiation of long-term passaged myoblasts across background strains compared to the other substrates tested. Moreover, while MG differentiated well during BL10 expansion, both Dba and BL6 expansions lacked differentiation suggesting that laminin 521 expansion may provide more consistent differentiation following expansion.

### Initial expansion on ECM dictates differentiation on other substrates

**Fig. 4:**
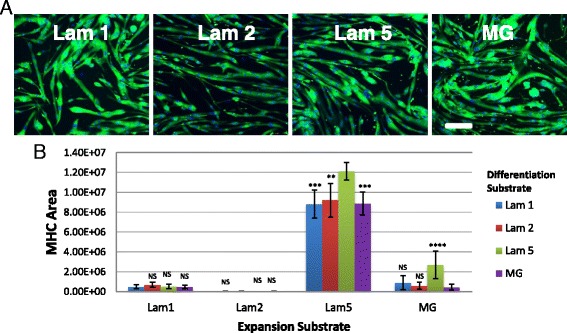
Expansion of Dba satellite cell-derived myoblasts on laminin 521 maintains differentiation when transferred to alternate substrates. Passage 8 myoblasts were transferred from laminin 521 to alternate substrates (**a**) but still show robust differentiation visualized by MHC expression (*green*) and Hoechst (*blue*). MHC area was quantified for all the different expansion and differentiation conditions (**b**). Laminin 521 expanded cells differentiated on all substrates. *Error bars* represent ±S.D. *Scale bar* represents 200 μM. Statistical significance was determined by one-way ANOVA with Bonferroni correction. *N* = 10 technical replicates from one biological sample. Significance is annotated as less than .01 (**), less than .001 (***), and less than .0001 (****)

To determine the utility of using laminin 521 for cell types already expanded on substrates other than laminin 521, we performed a substrate substitution experiment evaluating our previously isolated primary mouse myoblasts on each of the other substrates in our study. Dba cells expanded on laminin 521 show robust differentiation when transferred to any of the substrates tested here including laminin 111, laminin 211, and MG (Fig. 4a, b). In comparison, while cells maintained on laminin 521 demonstrated the highest differentiation performance, cells moved from laminin 521 to other substrates (laminin 111, laminin 211, and MG) showed a small reduction in differentiation (Fig. 4a, b). Additionally, we observed a lag in the initial proliferation when laminin 521 cells were transferred to other substrates, although cultures did gradually increase proliferation over time (data not shown). The cells expanded on all other substrates, including laminin 111, laminin 211, and MG and failed to differentiate significantly, both on their original substrates and when moved to other substrates (Fig. 4b). These results reveal that laminin 521 expanded myoblasts demonstrate superior propensity to maintain differentiation and reveal unique, broad, substrate transfer compatibility.

**Fig. 5:**
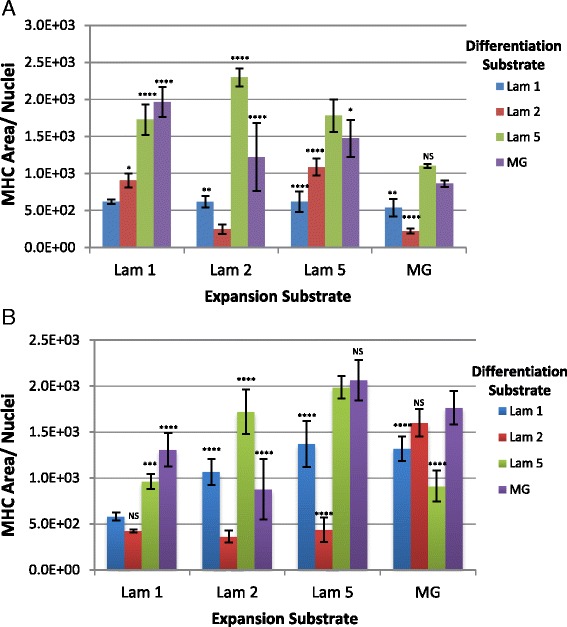
Long-term expanded BL6 and BL10 myoblasts transferred to alternative substrates show variable responses. Differentiation was quantified by calculating MHC area in proportion to total nuclei for BL6 (**a**) and BL10 (**b**) passage 8 myoblasts. Statistical significance was determined by one-way ANOVA with Bonferroni correction. *N* = 6 technical replicates. Significance is annotated as less than .05 (*), less than .01 (**), less than .001 (***), and less than .0001 (****)

Next, we repeated the ECM switching experiments in our long-term BL6 and BL10 cultures to determine if we observe similar substrate compatibility on different backgrounds. In BL6 and BL10 cultures, the cells expanded on laminin 111 increased their differentiation efficiency when moved to both laminin 521 and MG (Fig. 5a, b). However, laminin 211 expanded BL6 and BL10 cells showed the most significant increase in differentiation on laminin 521 (Fig. 5a, b). Interestingly, laminin 521 expanded BL6 cells moved to laminin 211 and MG differentiated similarly to control but transferring to laminin 111 showed a marked decrease in differentiation (Fig. 5a). Moreover, laminin 521 expanded BL10 cells moved to laminin 111, and MG showed similar differentiation compared to the control while cells moved to laminin 211 displayed a drop in differentiation (Fig. 5b). BL6 MG expanded cells moved to laminin 521 for differentiation maintained similar performance compared to the control while there was a significant reduction in differentiation when cells were transferred to laminin 111 or laminin 211 (Fig. 5a). In BL10 MG expanded cultures, the cells transferred to laminin 111 and laminin 211 differentiated similar to the control but the cells moved to laminin 521 displayed a decrease in differentiation (Fig. 5b). Overall, these results demonstrate that both laminin 521 and MG can increase the differentiation performance of myogenic cells previously expanded on different matrices. However, we observe significant differences in the compatibility and performance gains or losses that vary with the specific substrates and background used.

### Expanded myoblasts express similar myogenic markers across matrices

**Fig. 6:**
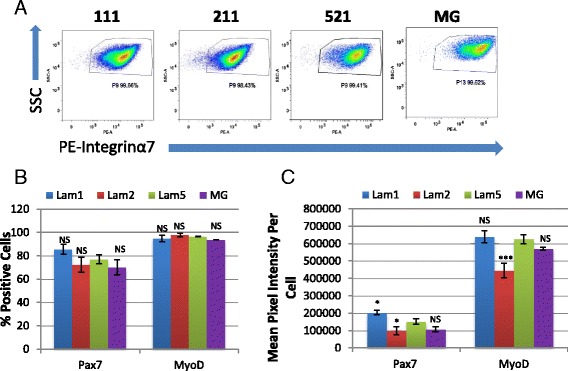
Expanded cells are purely myogenic and exhibit similar Pax7/MyoD expression. **a** Cells display greater than 99% positive expression of satellite cell/myoblast marker integrin α7—PE. Twenty thousand cells were analyzed for each substrate. **b**, **c** Cells on all substrates maintain similar expression regarding proportion of positive cells and staining intensity for both Pax7 and MyoD. *N* = 6 technical replicates for each group from one biological sample. Significance was determined by one-way ANOVA with Bonferroni correction. Significance is annotated as less than .05 (*), and less than .001 (***). All comparisons were conducted using laminin 521 as control

Due to the large differences observed in myogenic cell performance, we performed a set of control experiments to rule out the presence of contaminating non-myogenic cells in our primary mouse myoblast cell cultures. FACS staining revealed 99% of cells stained positive for integrin α7 (Fig. 6a) while there were no detectable PDGFRα- or CD31-positive cells present (data not shown) suggesting that our cultures were homogenous for myogenic cells. Subsequently, we immunostained cells from passage 6, during the expansion phase in growth medium, to determine the expression of Pax7 and MyoD to assess if changes in their expression or intensity may explain the dramatic difference in myogenic activity on different ECMs (Fig. 6b, c). We observed similar proportions of Pax7 and MyoD positively stained cells on each substrate (Fig. 6b). Protein staining intensity level varied minimally for Pax7 and MyoD expression (Fig. 6c). We observed statistically significant differences for Pax7 expression level changes; however, changes were small ranging around ±50% compared to laminin 521 (Fig. 6c). MyoD expression levels were consistent except for a reduction in expression level in laminin 211 cultures. Interestingly, Sca1 expression was found to be present in a significant proportion of cells cultured on laminin 111 and laminin 211 but was absent in cultures maintained on laminin 521 and MG (data not shown). These results agree with previous studies in which case Sca1 expression on myoblasts was associated with cells exhibiting poor differentiation [22]. These results suggest that Sca1 may be a marker associated with differentiation-deficient myogenic cells; however, this will require further study.

### FACS profiling reveals variation in integrin expression

Integrin receptor signaling plays many critical roles during myogenesis. Since laminins and components of MG activate many of their functions via integrin receptors, we hypothesized that our observed differences in long-term culture may be caused by shifts in integrin expression on different ECMs. Previously expanded Dba mouse cells were assayed at passage 8 in growth conditions on each ECM (laminin 111, laminin 211, laminin 521, and MG) by FACS staining using integrin α1–7, integrin αV, and integrin β1–5 antibodies (Table 1).

**Table 1:** Integrin FACS profiling on passage 8 Dba cells

	111	211	521	MG
α1	22.13	12.45	25.95	19.61
α2	0.55	0.19	2.63	5.41
α3	5.59	12.73	30.35	34.41
α4	67.53	42.86	67.64	90.98
α5	99.05	92.36	80.46	93.86
α6	78.81	67.66	62.4	96.61
α7	98.92	97.4	94.48	99.84
β1	99.01	99.07	96.04	99.95
β2	88.11	53.92	62.3	82.72
β3	69.59	67.05	44.08	57.48
β4	35.58	1.52	44.8	85.72
β5	0.2	0.17	0.57	3.94

Values represent % cells positive for selected integrins on Dba passage cells

Close to 100% of cells grown on all substrates expressed integrin α7 and β1 (Table 1). On the other hand, only a small proportion of cells expressed α1 (10–20%) while less than 5% of cells on any substrate expressed α2 or β5 (Table 1). Meanwhile, the remaining integrins showed some degree of heterogeneous expression across substrates. Expression of α4, α5, β2, and β4 was similar and heterogeneous on most substrates, with the exception of the high expression of β2 on laminin 111 and MG, and an absence of expression of β4 on laminin 211 and elevated expression on MG (Table 1). Most intriguingly, integrin α3 was expressed by a larger proportion of cells, approximately 30%, on cells expanded on laminin 521 and MG while it was expressed by less than 10% on cells expanded on all other ECMs (Table 1). In addition to population changes, we also observed variations in mean fluorescent intensities for a subset of integrins including integrin α3, integrin α5, integrin α6, integrin α7, integrin β2, and integrin β4 (Table 2). For example, integrin α3 showed elevated expression on laminin 521 expanded cells. Integrin α5 showed elevated expression in cells grown on laminin 111 and laminin 211, while integrin β2 had highest expression on laminin 111 (Table 2). Integrin α6 expression was increased dramatically on MG cultured cells while integrin α7 was expressed higher on MG cultured cells and, to a lesser extent, on laminin 521 cells (Table 2). Lastly, integrin β4 showed very little expression on laminin 211 cells while it was up-regulated in MG cultured cells (Table 2). Taken together, these results suggest that both the proportion of cells expressing each integrin and the expression level of integrins varies with different ECM matrices. Moreover, due to the complexity observed here, there are likely multiple mechanisms contributing to the different characteristics of cells expanded on different ECMs.

**Table 2:** Mean fluorescent intensity on passage 8 Dba cells

	111	211	521	MG
α3	2436	2848	3704	2167
α5	344,468	377,705	111,181	46,012
α6	4418	4316	5556	43,222
α7	21,973	23,195	44,471	73,179
β2	25,736	4909	5829	5772
β4	1890	601	3217	7345

### Laminin 521 supports enhanced human myoblast expansion and differentiation

**Fig. 7:**
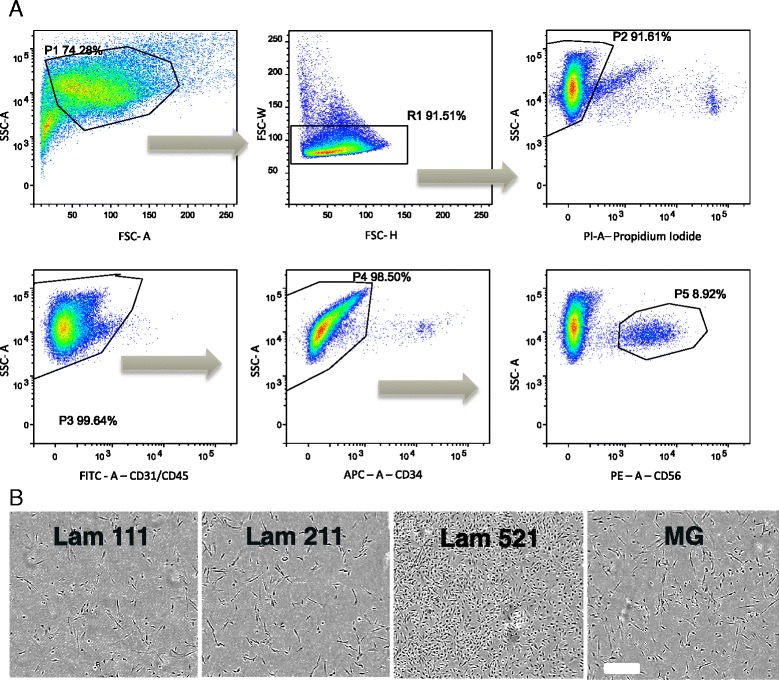
FACS isolation and early growth of human satellite cells. **a** FACS isolation for human satellite cells selecting for granularity (SSC × FSC), singlet selection (FSC-W × FSC-H, SSC-W × SSC-H), live/dead (propidium iodide), CD31−/CD45− (FITC), CD34− (APC), and CD56+ (PE). **b** Laminin 521 shows a clear growth advantage over all other tested substrates. *Scale bar* represents 300 μM

In an effort to determine if the result obtained with the mouse model translated to humans, we identified a source of freshly isolated human muscle and performed a similar evaluation. Human satellite cells were isolated from the gracillis muscle obtained from a post-mortem patient lacking diagnosed skeletal muscle disease. CD56+/CD31−/CD45−/CD11b− satellite cells were isolated using a dual MACS/FACS approach (Fig. 7a). The cells were initially expanded on each of the following substrates: laminin 111, laminin 211, laminin 521, and MG. Within the first week of growth, we observed a dramatically increased growth rate with cells expanded on laminin 521 compared to all other substrates (Fig. 7b). Additionally, laminin 211 appeared to display a lag in cell growth compared to other substrates, similar to our earlier satellite cell findings in mouse (Fig. 7b).

**Fig. 8:**
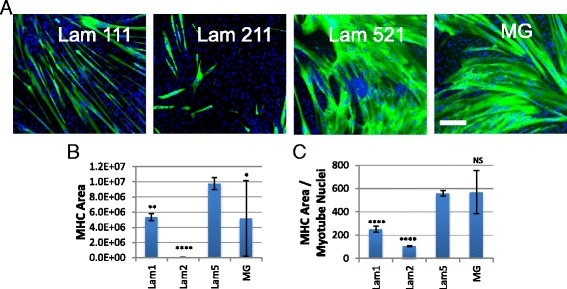
Laminin 521 supports improved human myogenic cell differentiation. **a** Cells expanded on laminin 521 and MG show significant differentiation after five passages in vitro, MHC (*green*), Hoechst (*blue*). **b** MHC area quantification shows increased differentiation in laminin 521 cultures and better stability compared to MG. **c** MHC/nuclear ratios show laminin 521 and MG differentiated cells display hypertrophy. *Error bars* represent ±S.D. *Scale bar* represents 200 μm. *N* = 10 technical replicates from one biological source. Significance was determined by one-way ANOVA with Bonferroni correction. Significance is annotated as less than .05 (*), less than .01 (**), and less than .0001 (****). All comparisons were conducted using laminin 521 as control

Since our mouse studies revealed unexpected expansion and differentiation effects on long-term culture of satellite cell-derived myoblasts on specific substrates, we next performed a similar study on our newly generated human myogenic cells. Human myoblasts expanded at a faster rate on laminin 521 compared to other substrates following five passages (Additional file 3: Figure S3). In addition, the human cells displayed the highest amount of differentiation on laminin 521 and MG, followed by moderate differentiation on laminin 111 and poor differentiation on laminin 211 (Fig. 8a, b). In addition, myotubes formed on laminin 521 and MG appeared to be hypertrophic due to an increased amount of MHC area staining in proportion to myotube nuclear count (Fig. 8c). Importantly, we observed differentiated myotubes maintained better attachment on laminin 521 compared to MG, in which case we observed larger variability on MG due to myotube detachment. Laminin 211-differentiated cultures performed poorly, similar to the observations with the mouse cultures. This resulted in a majority of cells staining negative for MHC expression and a very small MHC area value (Fig. 8b, c). Laminin 111 differentiated well but only reached approximately half of the MHC area compared to laminin 521 or MG cultures (Fig. 8b).

## Discussion

A vast amount of research has been performed on satellite cell/myoblast biology and myogenesis over the past 50 years. This work has yielded an enormous depth of knowledge encompassing wide number of molecular regulators ranging from transcription factors, signaling molecules, and ECM proteins. Despite this progress, there remains a significant disparity in work to support cell therapy and drug discovery efforts with satellite cells and myoblasts. A number of challenges have remained unsolved; how to scale up a relatively small number of skeletal muscle precursors into hundreds of millions or billions of cells for high-throughput drug screening or stem cell engraftment. In order to perform drug discovery in relevant models derived from stem cells isolated from patients, it is critical that the cells maintain the expansion and differentiation potential of the primary cell type. In addition, while Matrigel has become a very common ECM for satellite cell and myoblast culture, it is critical to establish an alternative ECM substrate which is animal-free and non-xenogeneic for cell therapy development and clinical trials. Given that target cells for assays addressing the majority of neuromuscular disease are multinucleated and differentiated myotubes and not transit-amplifying myogenic cells, it is critical that culture conditions support the ability of the expanded primary cells to differentiate effectively into myotubes. Many of the common reagents used for basic biology research, particularly ECM substrates, have not been tested in long-term experiments with multi-passage myogenic cell cultures. Because of this disparity, it is crucial to perform culture testing over the long term; doing so will help advance both basic muscle research and accelerate drug development advancement by broadly improving myogenic cell performance. Here, we provide the first comprehensive analysis detailing the proliferation and differentiation performance of different ECM’s on both mouse and human myogenic cells in short-term and long-term expansion.

The studies reported here demonstrate that the optimal ECM substrates for large-scale myogenic expansion are those that incorporate laminin α5, a previously underappreciated isoform of the laminin family in skeletal muscle. While laminin α5 is normally expressed during regeneration in both Ctx mouse models and DMD tissue, no study has been performed regarding its utility in in vitro myogenesis studies [10]. Here, our mouse studies show laminin 521 (α5, β2, λ1) supports superior myoblast performance in vitro by facilitating impressive proliferation and maintenance of differentiation after eight passages and approximately a 5000-fold expansion. These results indicate that laminin 521 may be a more ideal substrate for large scale-up applications, by helping to minimize the loss in differentiation potential over time. In our studies, mouse myogenic cells expanded on laminin 111 progressively lost their ability to up-regulate MHC while MG-expanded cells entered a state whereby they entered a myocyte stage expressing MHC but then fail to fuse. In addition to the mouse study presented here, we have performed an analysis of freshly isolated mdx/BL10 cells, and we observed a very similar pattern with laminin 521 outperforming laminin 111, laminin 211, and MG in differentiation (Additional file 4: Figure S4). Furthermore, we have encountered difficulty in expanding mdx/BL10 cells on laminin 111 while we have not encountered these issues on laminin 521 (data not shown). Importantly, our findings translate to human myogenic cell culture as our human cells perform optimally on laminin 521 showing superior proliferation and differentiation to all other substrates tested. Taken together, our results demonstrate laminin 521 as an optimal substrate for myoblast expansion while demonstrating translatability across several mouse backgrounds (Dba/2J, C57/BL10, C57/BL6), human cells, and disease states (mdx/BL10).

While our results strongly support laminin 521 as a superior myoblast culture matrix, additional studies will be required to further evaluate the effects of laminin 521. Particularly, additional study will need to be conducted with additional human samples to confirm that laminin 521 is broadly beneficial across multiple human myogenic progenitors. We have directly measured the amount of protein coating among the different substrates used in this study and find only a minimal difference between the different laminins and gelatin used in our study (Additional file 5: Figure S5). Matrigel resulted in an increased amount of protein compared to other substrates (Additional file 5: Figure S5). Despite these differences in coating amounts, we do not observe significant differences in cellular adherence in either freshly isolated cells (Additional file 6: Figure S6A) or long-term passaged cells (Additional file 6: Figure S6B). We have observed however that the presence of high concentrations of calcium and magnesium, as found in HBSS and PBS containing calcium and magnesium, are critical for laminin coating function. The inclusion of calcium and magnesium during laminin coating is not commonly included in many of the commercial vendor protocols, despite the critical roles of calcium and magnesium in laminin signaling.

The observed benefits of laminin 521 are likely due to the larger number of integrin binding sights on laminin α5 including unique binding sites for integrin α3, αV, and α6β4 not present in other laminin isoforms [12–15]. Laminin 521 can therefore be seen as a richer substrate capable of interacting with six integrin binding sites (α3β1 (2×), αVβ3, α6β1, α6β4, α7β1), compared to four binding sites in laminin 111 and MG (α1β1, α2β1, α6β1, α7β1) [12, 15]. The converse also may be true for laminin 211; it may fail to stimulate a high level of proliferation due to the presence of only three integrin-binding sites (α1β1, α2β1, α7β1) [12]. Our studies have shown that a greater proportion of cells express integrin α3 on laminin 521 and MG expanded cells compared to all other substrates (Table 1). Multiple studies show that integrin α3 is a critical component during myogenic cell differentiation [23, 24]. However, our results also suggest that integrin α3-laminin α5 interaction may not be critical for differentiation since cells moved from laminin 521 to other substrates maintain the ability to differentiate, even on the minimalistic laminin 211. While our studies show MG expanded cells turn on the differentiation program through up-regulation of MHC, there is a deficit in myoblast fusion in long-term cultures. We observed an increased expression level of integrin α7 in both laminin 521 and MG cultures (Fig. 6). Since integrin α7 increases myoblast mobility and is dramatically increased during myogenic differentiation, increased expression of integrin α7 in our cultures is a possible mechanism promoting enhanced differentiation. These findings may suggest that expression of integrin α3 and high expression of integrin α7 assist cells in initiating the differentiation program but additional mechanisms are likely responsible for the difference in myotube formation between cells expanded on laminin 521 and MG. Other key differences observed included decreased expression level of integrin α5 on laminin 521 expanded cells and increased proportions of cells expressing integrin β2 and integrin β4 in MG cultures. Studies have shown expression of integrin α5 regulates myogenesis by favoring expansion and inhibiting differentiation, thus reduced integrin α5 on laminin 521 expanded cells may contribute to the maintenance of differentiation in the long term [11, 25, 26]. Overall, while our studies reveal multiple differences in the expression of integrins following long-term culture, however, there appears to be some long-lasting effects of growth on each of the ECM substrates that is poorly understood and cannot be explained merely by mapping integrin-ECM binding interactions. It will be critical in future experiments to use integrin inhibition to determine which integrins are critical for maintaining differentiation ability in myogenic cultures. While we acknowledge our integrin study is limited due to the inclusion of only the Dba mouse strain, most importantly, our findings encourage future investigation of integrin expression across backgrounds, species, and passage time points in culture. It will be crucial to expand on these findings as we expect integrin expression to vary between biological samples and conditions.

This study significantly demonstrates that various substrates are not created equal when they are employed for the expansion of myoblasts in vitro. While the performance of laminin 521 is impressive, there may be room for improvement by combining additional ECMs with laminin 521. First, it will be important to analyze the combination of laminin 521 with laminin 111 and laminin 211 to determine if there is any potential synergism. While laminin 521 fared the best in differentiation after scale-up, spontaneous differentiation was still present at a low level. Collagen VI has been described as a critical mediator of stem cell renewal in the satellite cell niche suggesting it may prevent progression into differentiation [27]. Considering this, collagen VI in combination with laminin 521 may reduce the spontaneous differentiation during expansion, at which time collagen VI could be removed to induce differentiation. ECM stiffness is a critical regulator balancing myogenesis towards proliferation or differentiation [28–32]. Combining laminin 521 with MG or a hydrogel may provide increased performance in both proliferation and/or differentiation by providing a method to customize stiffness of the substrate to optimize both proliferation and differentiation conditions. It will be important in future studies to titrate laminin 521, which will affect both substrate stiffness and geometry of the laminin matrix, both of which may significantly affect proliferation and differentiation.

Two of the most popular myogenic matrices routinely employed in stem cell biology are MG and laminin 111; however, neither of these substrates is endogenous to the satellite cell niche. Additionally, MG is a highly variable substrate isolated from mouse EHS tumors containing over 14,000 peptides resulting in experimental variability and decreased reproducibility [33]. In fact, even growth factor reduced MG, as used in this study, contains over 9000 peptides and significant variation as high as 50% from batch to batch has been observed which makes cell culture performance inconsistent [33]. The laminin α1 chain contained in both MG and laminin 111 is absent from adult skeletal muscle in both resting and regenerating conditions and is only expressed in a small stage of developmental myogenesis [10, 34, 35]. This discrepancy has by and large gone unverified with the assumption that laminin 111 and laminin 211 share redundant functionality. For instance, pre-clinical animal studies have been performed injecting laminin 111 into dystrophic mice despite the lack of α1 in the muscle [36]. However, these results are controversial since follow-up transgenic laminin 111 studies show no benefit in the *mdx* model [37]. Our results show for the first time that there are significant functional differences between the laminin α1 and α2 chains. While α1 supports moderate proliferation and differentiation in our studies, α2 tends to limit proliferation and supports differentiation poorly. Considering this in combination with our results, laminin 511 or laminin 521 supplementation/overexpression may be superior therapeutically for muscular dystrophies including DMD since they contain the beneficial integrin α7 binding site similar to laminin 211 and laminin 111, but laminin 511 and laminin 521 may additionally boost the regenerative response. One caveat for potential intramuscular laminin α5 injection or overexpression is that laminin 521 is normally expressed at neuromuscular junctions while laminin 511 surrounds regenerating myofibers; thus, in vivo comparison studies between laminin 511 and laminin 521 will be crucial [10].

The studies presented here suggest that the laminin 521 findings may be translatable to additional technologies including cell therapy-engraftment technology and iPS skeletal muscle differentiation. In recent years, cell therapy for the muscular dystrophies has lost favor due to the inability to generate sufficient numbers of engraftment-capable myoblasts for transplantation. In the early 90s, while cell therapy was a highly promising technology due to newly developed myoblast cell culture, human myoblast trials were a failure due to the inability of the in vitro propagated cells to efficiently engraft or provide functional muscle strength recovery [38–41]. Subsequent mouse studies demonstrated that freshly isolated myogenic cells are highly efficient at engrafting, but over time, the culture engraftment potential dramatically falls [42]. Historically, cell therapy and engraftment experiments commonly utilize gelatin, MG, or laminin 111 as expansion substrates [21, 43–46]. As demonstrated in the results presented in this report, these are not the best substrates for in vitro scale up of pre-implanted cells. Our studies suggest that laminin 521 may provide a better option for the scale-up of cells for cell therapy since we demonstrate that laminin 521 is the only substrate in our studies capable of maintaining differentiation over an extended expansion period. Studies are currently underway to analyze the engraftment potential of mouse myoblasts expanded on varying substrates including laminin 521. The differentiation of skeletal muscle differentiation from iPSCs is currently not well defined even though a number of reports have been published using protocols with varying techniques and ECM substrates [47–50]. Since laminin α2, laminin α4, and laminin α5 are expressed during muscle development, it will be important to test these substrates in iPSC studies to determine the optimal ECM induction substrate [10, 11]. Additionally, while multiple protocols exist to produce iPSC-derived myogenic cells, iPSC-generated skeletal muscle cell expansion has not been well characterized examining performance after large scale-up of the generated cells. In this avenue, laminin 521 may provide a significant advantage in maintaining iPSC myogenic cells in a differentiation-competent state while the cells are expanded into large numbers for drug screening and cell therapy applications.

## Conclusions

Laminin 521 represents a new high performance, biologically relevant matrix for superior muscle cell performance in vitro. Laminin 521 supports generation of larger myotubes and higher amounts of nuclei per myotube compared to all other substrates with the exception of Matrigel. However, while laminin 521 matches the performance of Matrigel, it also provides more consistent and reliable differentiation over long-term culture. Particularly, laminin 521 represents an excellent substrate for cell therapy development and clinical trials due to its human origin; Matrigel on the other hand is not compatible with clinical trials or cell therapy applications in humans. Laminin 521 appears to increase differentiation potentially without altering the traditional Pax7/MyoD paradigm; it is likely that laminin 521 is providing novel cell regulation not previously appreciated. It may be beneficial to integrate laminin 521 into currently established techniques in muscle biology research to achieve synergism with other matrix proteins or technologies. In conclusion, laminin 521 extends the use of isolated primary cells and is likely to enhance many applications requiring large growth expansion such as drug discovery, genetic engineering, and cell therapy.

## Additional files

Additional file 1: Figure S1. Laminin chain structure. Laminins are heterotrimeric complexes annotated in the order of α, β, and λ chains. Examples are laminin 111 = α1, β1, and λ1 and laminin 521 = α5, β2, and λ1.
Additional file 2: Figure S2. FACS sort of DBA/2J satellite cells. Cells were sorted for granularity (SSC × FSC), singlet selection (FSC-W × FSC-H, SSC-W × SSC-H), live/dead (propidium iodide), CD31−/CD45− (Percp-eFluor710), Pdgfrα−/Sca1− (BV421/BV605), and integrin α7+ (PE).
Additional file 3: Figure S3. Laminin 521 promotes increased human myogenic cell proliferation. Cells were expanded on laminin 111, laminin 211, laminin 521, or growth factor reduced Matrigel for 5 passages. For proliferation analysis, cells were seeded at a density of approximately 1500 cells per well and incubated for 2 days. Afterwards, cells were fixed and nuclei counts were performed using Hoechst staining (Life Technologies). *N* = 5 technical replicates. Significance was determined by one-way ANOVA with Bonferroni correction. Significance is annotated as less than .0001 (****) with laminin 521 as control.
Additional file 4: Figure S4. Laminin 521 promotes increased mdx/BL10 differentiation. Cells were freshly isolated from male mdx/BL10 limb muscles and plated on multiple matrices: laminin 111, laminin 211, laminin 521, and growth factor reduced Matrigel. MHC area quantification shows a trend towards increased differentiation on laminin 521 compared to other substrates. *N* = 3 technical replicates per substrate. Differences were not found to be statistically different by one-way ANOVA and Bonferroni correction.
Additional file 5: Figure S5. Quantification of protein coating for various ECM proteins. Laminin 111, laminin 211, laminin 521, and gelatin coat similarly, while Matrigel is coated at a higher amount. *N* = 12 technical replicates. Data is presented as mean with error bars representing standard deviation. Statistics determined by one-way ANOVA with Bonferroni correction. Significance is annotated as less than .001 (***) using laminin 521 as control.
Additional file 6: Figure S6. Cell counts for freshly isolated and expanded myoblasts reveal similar cell adherence across all matrices. (A) Cell counts from freshly isolated Dba, BL6, and BL10 cells 5 days post isolation show similar cell adherence on all matrices. (B) Cell counts for Dba, BL6, and BL10 myoblasts after passage 8 (8 h post plating) reveal similar cell adherence on all matrices following long-term culture. *N* = 3 biological replicates (1 each for BL6, Bl10, and Dba). Differences were not found to be statistically different by one-way ANOVA and Bonferroni correction. Mean column includes standard deviation values.

## Abbreviations

ECM: Extracellular matrix
FACS: Fluorescent-activated cell sorting
FN: Fibronectin
GFR-MG: Growth factor reduced Matrigel
IPSC: Induced pluripotent stem cell
LAM: Laminin
MG: Matrigel
MHC: Myosin heavy chain
SC: Satellite cell
