# Supplementary figures and images for: Laminin 521 maintains differentiation potential of mouse and human satellite cell-derived myoblasts during long-term culture expansion
Source: Skelet Muscle. 2016 Dec 13;6:44. doi: 10.1186/s13395-016-0116-4 (PMC5154152; doi:10.1186/s13395-016-0116-4)

## Slide 1
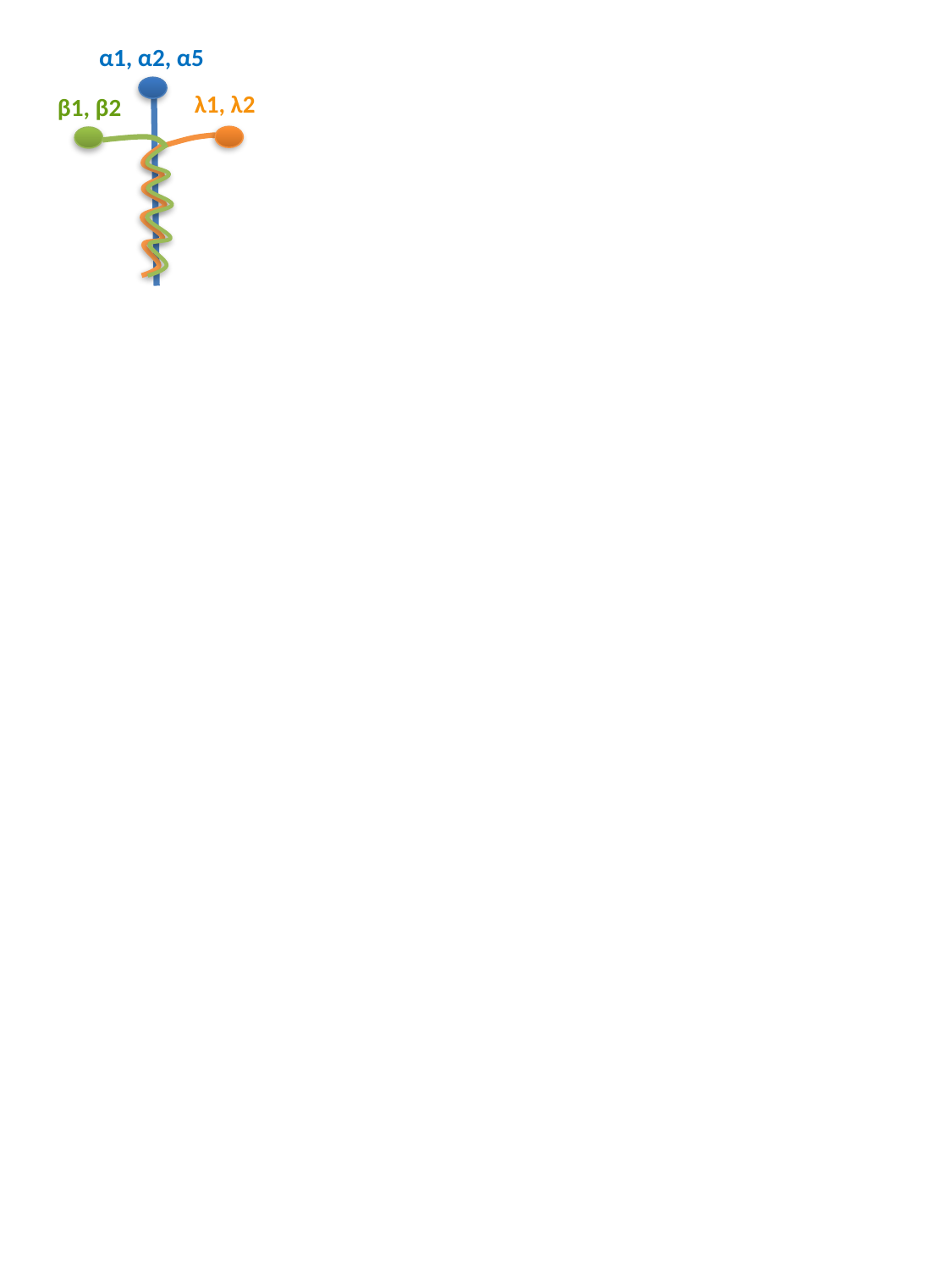

α1, α2, α5
λ1, λ2
β1, β2

Supplement: Additional file 1: Figure S1. — Laminin chain structure. Laminins are heterotrimeric complexes annotated in the order of α, β, and λ chains. Examples are laminin 111 = α1, β1, and λ1 and laminin 521 = α5, β2, and λ1. [file 13395_2016_116_MOESM1_ESM.pptx]

## Slide 1
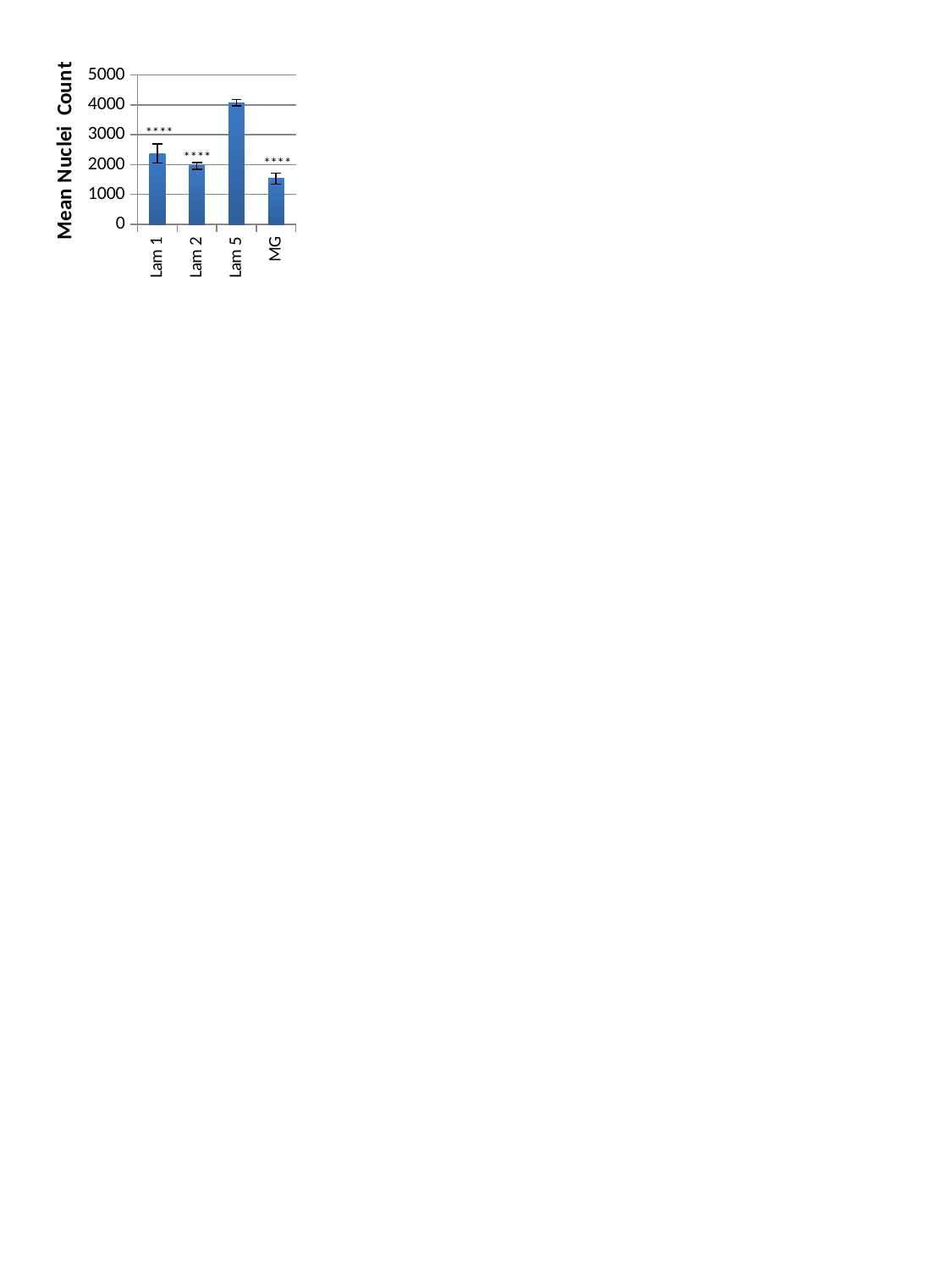

### Chart
| Category | |
|---|---|
| Lam 1 | 2373.8333333333335 |
| Lam 2 | 1958.1666666666667 |
| Lam 5 | 4074.3333333333335 |
| MG | 1534.3333333333333 |****
****
****

Supplement: Additional file 3: Figure S3. — Laminin 521 promotes increased human myogenic cell proliferation. Cells were expanded on laminin 111, laminin 211, laminin 521, or growth factor reduced Matrigel for 5 passages. For proliferation analysis, cells were seeded at a density of approximately 1500 cells per well and incubated for 2 days. Afterwards, cells were fixed and nuclei counts were performed using Hoechst staining (Life Technologies). N = 5 technical replicates. Significance was determined by one-way ANOVA with Bonferroni correction. Significance is annotated as less than .0001 (****) with laminin 521 as control. [file 13395_2016_116_MOESM3_ESM.pptx]

## Slide 1
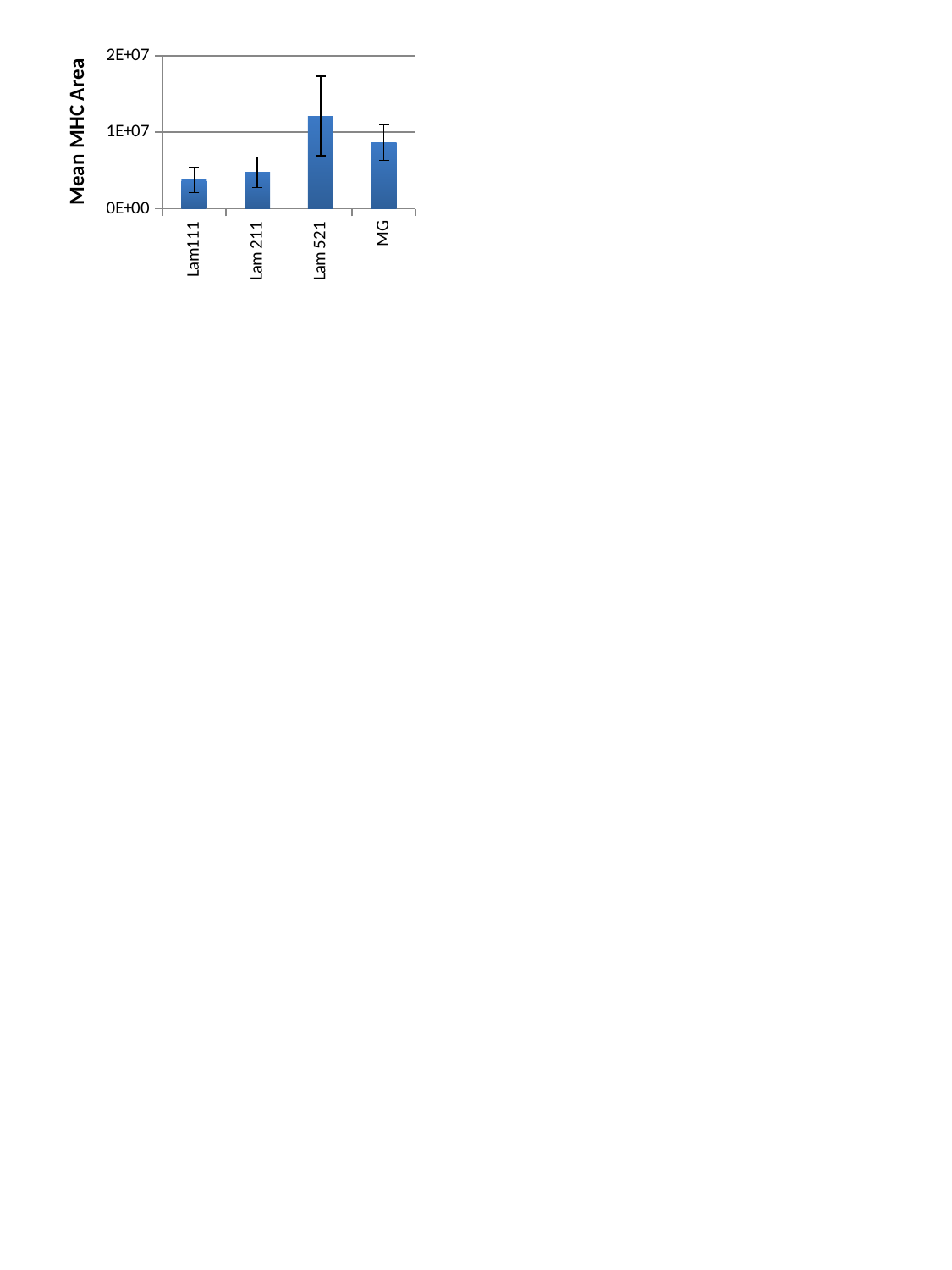

### Chart
| Category | |
|---|---|
| Lam111 | 3740137.2653333335 |
| Lam 211 | 4753419.021333333 |
| Lam 521 | 12110548.873000002 |
| MG | 8674042.185 |

Supplement: Additional file 4: Figure S4. — Laminin 521 promotes increased mdx/BL10 differentiation. Cells were freshly isolated from male mdx/BL10 limb muscles and plated on multiple matrices: laminin 111, laminin 211, laminin 521, and growth factor reduced Matrigel. MHC area quantification shows a trend towards increased differentiation on laminin 521 compared to other substrates. N = 3 technical replicates per substrate. Differences were not found to be statistically different by one-way ANOVA and Bonferroni correction. [file 13395_2016_116_MOESM4_ESM.pptx]

## Slide 1
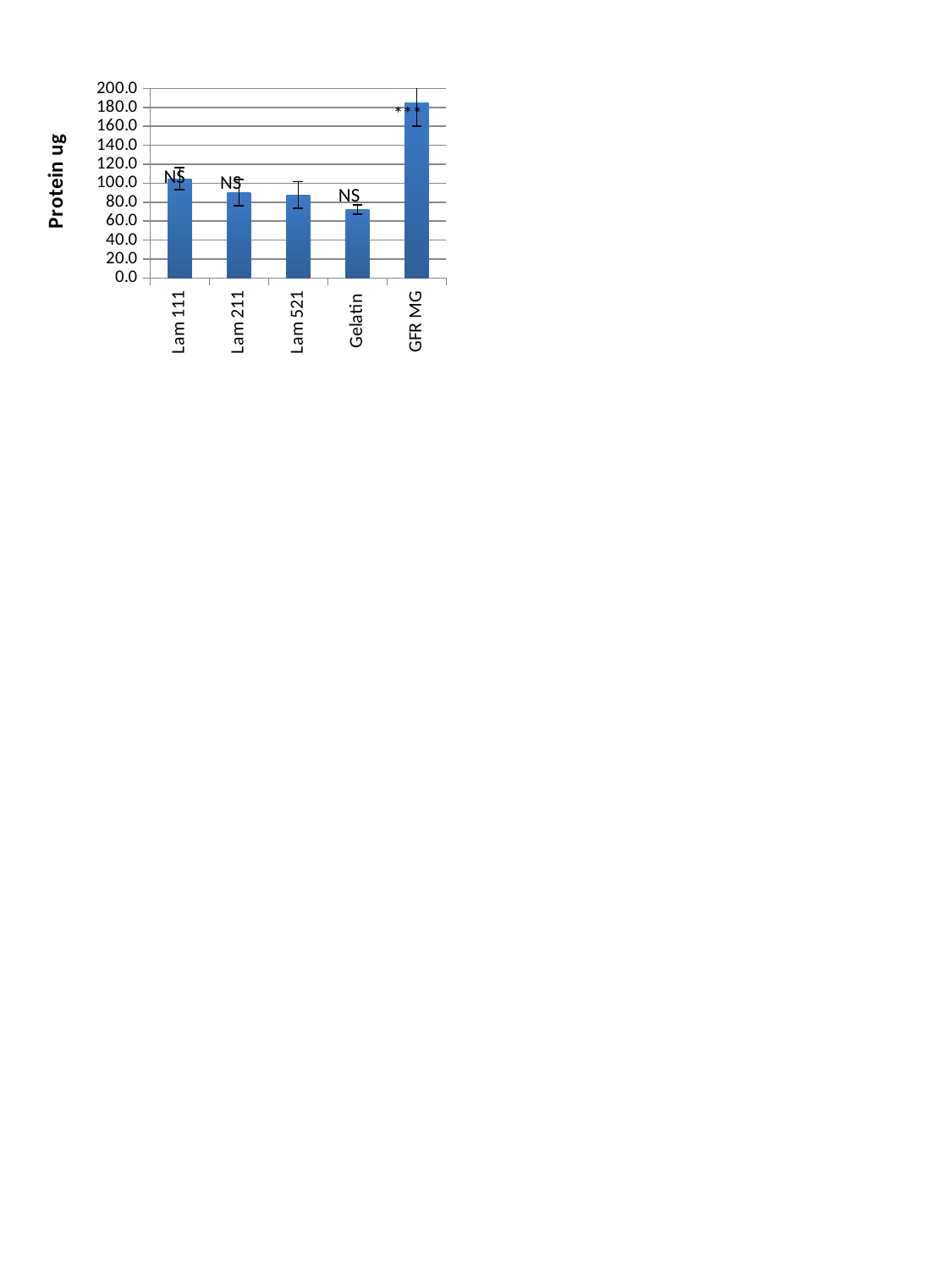

### Chart
| Category | |
|---|---|
| Lam 111 | 104.71773749999998 |
| Lam 211 | 90.22916136363635 |
| Lam 521 | 87.74628125 |
| Gelatin | 72.39650624999999 |
| GFR MG | 184.42096249999992 |***
NS
NS
NS

Supplement: Additional file 5: Figure S5. — Quantification of protein coating for various ECM proteins. Laminin 111, laminin 211, laminin 521, and gelatin coat similarly, while Matrigel is coated at a higher amount. N = 12 technical replicates. Data is presented as mean with error bars representing standard deviation. Statistics determined by one-way ANOVA with Bonferroni correction. Significance is annotated as less than .001 (***) using laminin 521 as control. [file 13395_2016_116_MOESM5_ESM.pptx]

## Slide 1
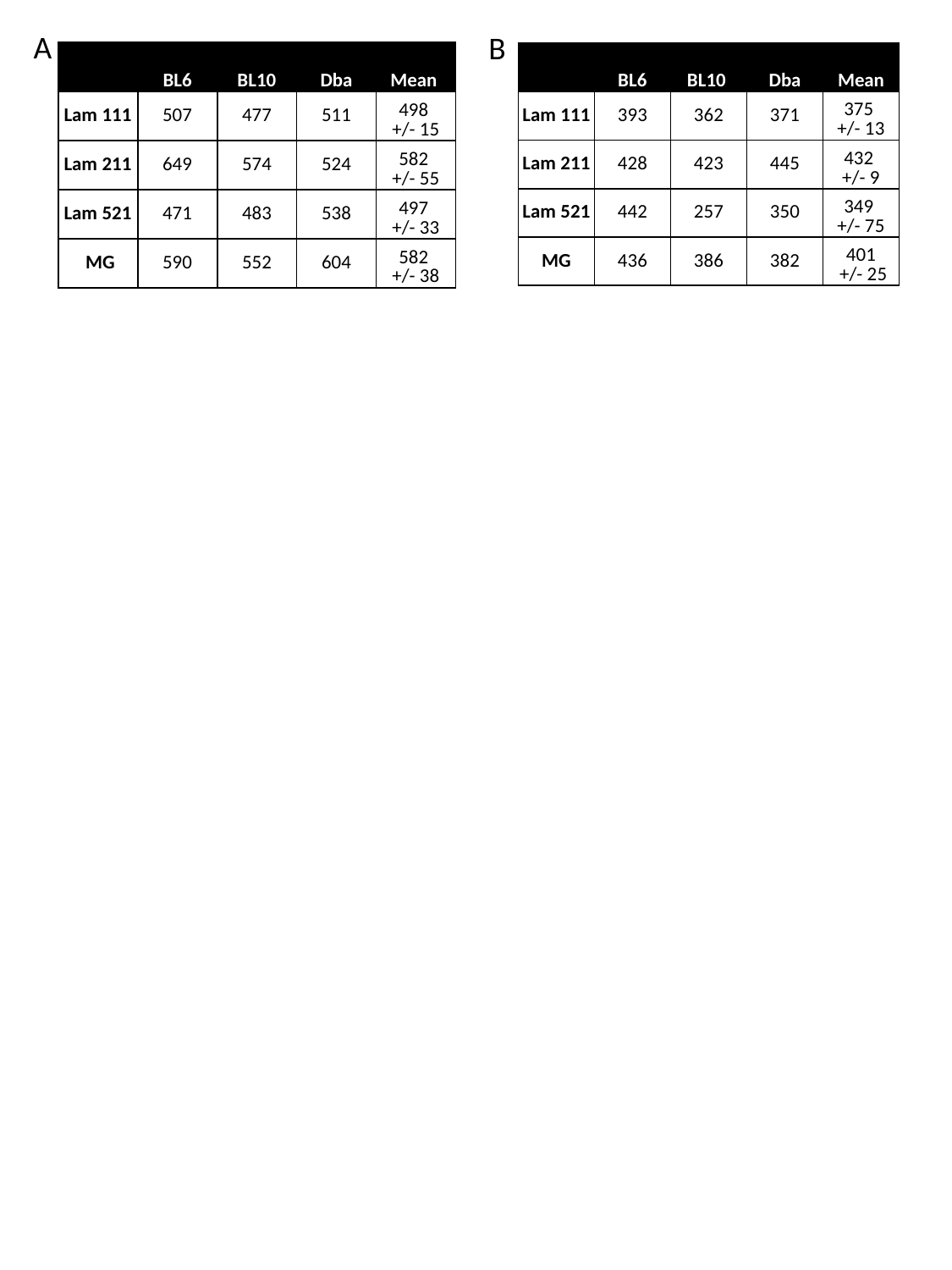

A
B
| | BL6 | BL10 | Dba | Mean |
| --- | --- | --- | --- | --- |
| Lam 111 | 507 | 477 | 511 | 498 +/- 15 |
| Lam 211 | 649 | 574 | 524 | 582 +/- 55 |
| Lam 521 | 471 | 483 | 538 | 497 +/- 33 |
| MG | 590 | 552 | 604 | 582 +/- 38 |
| | BL6 | BL10 | Dba | Mean |
| --- | --- | --- | --- | --- |
| Lam 111 | 393 | 362 | 371 | 375 +/- 13 |
| Lam 211 | 428 | 423 | 445 | 432 +/- 9 |
| Lam 521 | 442 | 257 | 350 | 349 +/- 75 |
| MG | 436 | 386 | 382 | 401 +/- 25 |

Supplement: Additional file 6: Figure S6. — Cell counts for freshly isolated and expanded myoblasts reveal similar cell adherence across all matrices. (A) Cell counts from freshly isolated Dba, BL6, and BL10 cells 5 days post isolation show similar cell adherence on all matrices. (B) Cell counts for Dba, BL6, and BL10 myoblasts after passage 8 (8 h post plating) reveal similar cell adherence on all matrices following long-term culture. N = 3 biological replicates (1 each for BL6, Bl10, and Dba). Differences were not found to be statistically different by one-way ANOVA and Bonferroni correction. Mean column includes standard deviation values. [file 13395_2016_116_MOESM6_ESM.pptx]
