# Supplementary material for: Laminin 521 maintains differentiation potential of mouse and human satellite cell-derived myoblasts during long-term culture expansion
Source: Skelet Muscle. 2016 Dec 13;6:44. doi: 10.1186/s13395-016-0116-4 (PMC5154152; doi:10.1186/s13395-016-0116-4)
Supplement: Additional file 2: Figure S2. — FACS sort of DBA/2J satellite cells. Cells were sorted for granularity (SSC × FSC), singlet selection (FSC-W × FSC-H, SSC-W × SSC-H), live/dead (propidium iodide), CD31−/CD45− (Percp-eFluor710), Pdgfrα−/Sca1− (BV421/BV605), and integrin α7+ (PE). [file 13395_2016_116_MOESM2_ESM.pptx]

## Slide 1
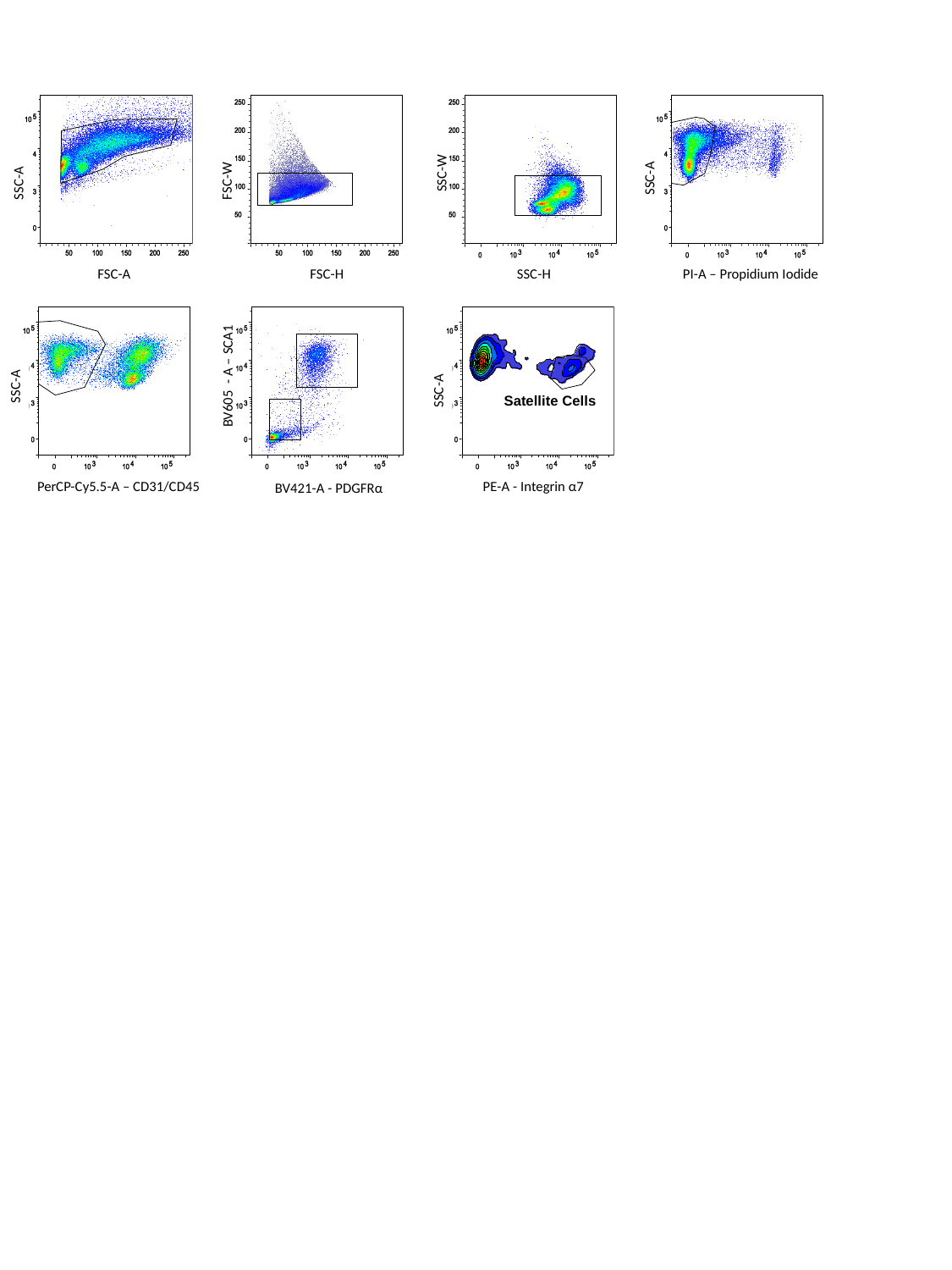

SSC-W
SSC-A
SSC-A
FSC-W
FSC-A
FSC-H
SSC-H
PI-A – Propidium Iodide
BV605 - A – SCA1
SSC-A
SSC-A
Satellite Cells
PerCP-Cy5.5-A – CD31/CD45
PE-A - Integrin α7
BV421-A - PDGFRα
